# Immunoglobulin Expression in Cancer Cells and Its Critical Roles in Tumorigenesis

**DOI:** 10.3389/fimmu.2021.613530

**Published:** 2021-03-24

**Authors:** Ming Cui, Jing Huang, Shenghua Zhang, Qiaofei Liu, Quan Liao, Xiaoyan Qiu

**Affiliations:** ^1^ Department of General Surgery, Peking Union Medical College Hospital, Chinese Academy of Medical Sciences, Peking Union Medical College, Beijing, China; ^2^ Department of Immunology, School of Basic Medical Sciences, Peking University, Beijing, China

**Keywords:** immunoglobulin, cancer-derived Ig, IgG, glycosylation, immune escape, metastasis

## Abstract

Traditionally, immunoglobulin (Ig) was believed to be produced by only B-lineage cells. However, increasing evidence has revealed a high level of Ig expression in cancer cells, and this Ig is named cancer-derived Ig. Further studies have shown that cancer-derived Ig shares identical basic structures with B cell-derived Ig but exhibits several distinct characteristics, including restricted variable region sequences and aberrant glycosylation. In contrast to B cell-derived Ig, which functions as an antibody in the humoral immune response, cancer-derived Ig exerts profound protumorigenic effects *via* multiple mechanisms, including promoting the malignant behaviors of cancer cells, mediating tumor immune escape, inducing inflammation, and activating the aggregation of platelets. Importantly, cancer-derived Ig shows promising potential for application as a diagnostic and therapeutic target in cancer patients. In this review, we summarize progress in the research area of cancer-derived Ig and discuss the perspectives of applying this novel target for the management of cancer patients.

## Introduction

Immunoglobulin (Ig) molecules comprise two immunoglobulin heavy (IgH) chains and two immunoglobulin light (IgL) chains, which are linked by disulfide bridges to form a structure with twofold symmetry. IgH chains are classified into five isotypes, namely, Igμ, Igγ, Igα, Igδ and Igϵ, four subclasses of Igγ (Igγ1, Igγ2, Igγ3 and Igγ4) and two subclasses of Igα (Igα1 and Igα2). IgL chains have the following two isotypes: Igκ and Igλ. The N-terminal regions of the IgH and IgL chains, which are known as variable (V) regions, are highly variable in their sequences and are responsible for antigen recognition. In contrast, the C-terminal regions of the IgH and IgL chains are constant in their sequences and, thus, are called constant (C) regions. The generation of V region diversity is essential for functional Ig expression and the ability of Ig molecules to recognize various antigens. Multiple molecular mechanisms participate in this process, including V(D)J recombination, somatic hypermutation (SHM) and class-switch recombination (CSR) (1, 2). Ig molecules are important components of humoral immune responses. These molecules function as antibodies in immune defense by neutralizing antigens and mediating antibody-dependent cellular cytotoxicity (ADCC), complement-dependent cytotoxicity (CDC) and antibody-dependent cellular phagocytosis (ADCP) (3, 4).

In patients with cancer, elevated levels of Ig molecules and monoclonal gammopathy were identified decades ago (5). These Ig molecules were considered tumor-reactive antibodies but seemed to be indicative of malignancy progression rather than defense functions. Since Ig was believed to be solely expressed by B lymphocytes, these phenomena were paradoxically interpreted as the “rebel” roles of B lymphocytes in antitumor immunity.

In 1996, the existence of an “Ig-like” protein was unexpectedly identified using anti-human IgG antibody and Protein A in several types of cancer cells (6). Due to the classic theory that Ig is produced by only B lymphocytes, it was difficult to understand at that time whether this “Ig-like” protein was an Ig molecule or another protein sharing a common epitope with Ig. Subsequently, several research groups have started to explore this molecule. First, the transcripts of the IgH variable region and constant region were detected in several epithelial cancer cell lines (7). Then, the expression of IgG in human epithelial cancer cells was comprehensively confirmed at both the transcriptional and protein levels (8). To avoid contamination with B lymphocytes, laser capture microdissection and flow cytometry were used to purify cancer cells from cancer tissues, and the expression of IgG was further validated in long-term-cultured cancer cell lines. During almost the same period, other isotypes of Ig, such as Igκ (9), IgA (10), and IgM (11), were also found to be expressed in cancer cells. Thus, clearly, Ig can be expressed by cancer cells, and this Ig is named cancer-derived Ig. In this review, we summarize current research concerning cancer-derived Ig and its critical roles in tumorigenesis.

## Wide Expression of Ig in Cancer Cells

### Expression Profile of Ig in Cancers

Generally, cancer-derived Ig is mainly detected in epithelial cancer cells, including those of breast cancer (12–15), colon cancer (16, 17), cervical cancer (16, 18), lung cancer (18–20), laryngeal cancer (21), nasopharyngeal cancer (22), pancreatic cancer (23–25), liver cancer (16, 18), prostate cancer (26–28), oral cancer (29, 30), thyroid cancer (31), parathyroid cancer (32), esophageal cancer (33), gastric cancer (34), renal cancer (35), and bladder cancer (36). At the subcellular level, cancer-derived Ig is mainly located in the cytoplasm and on the cell membrane. Cancer-derived Ig can also be detected in secreted forms in the supernatant of cultured cancer cells (8, 10). Moreover, cancer-derived Ig is detected in a wide variety of soft tissue tumors including malignant fibrous histiocytomas, fibrosarcomas, and leiomyosarcomas (37). The expression of cancer-derived Ig in sarcomas was confirmed in the sarcoma cell lines A673, U-2 OS and HT1080 (38). In addition, cancer-derived Ig is frequently expressed in acute myeloid leukemia cells (39–42). Thus, cancer-derived Ig is validated to be widely expressed in different cancer lineages, including epithelial cancers, soft tissue tumors, and hematopoietic neoplasms. The IGHG1, IGHG2, IGHG3, and IGHG4 mRNA levels in tumor and peritumoral tissues from The Cancer Genome Atlas (TCGA) database and Genotype-Tissue Expression (GTEx) database are shown according to GEPIA (http://gepia.cancer-pku.cn/) (43) (**Supplementary Figure 1**). The detailed information of cancer-derived Ig expression in cancers is summarized in **Table 1**.

**Table 1:** Cancer-derived Ig expression in different types of malignancies.

Malignancy types	Detected Ig isotypes	Positive ratio in patients (%)	Positive cell lines	Biological functions	Clinical significance	References
Breast cancer	IgG	81	MCF-7, MDA-MB-231, SKBR3, T47D, ZR75-1, MDA-435	Promotes growth, promotes tumor immune escape	Histological subtype, metastasis, clinical stage	(6, 7, 11, 13, 15, 44–46)
	IgA	97	SKBR3, MCF-7, MDA-MB-231, Bcap37	NA	Lymph node metastasis	(7, 10–12, 14)
Lung cancer	IgG	66	A549, NCI-H520, SK-MES-1, Calu-6, H441	Promotes growth, promotes invasion and migration	Local invasion, tumor differentiation, lymph node metastasis, prognosis	(8, 18, 20, 46, 47)
Colon cancer	IgG	55	HT-29, LoVo, SW480, HCT116, SW1116	Promotes growth, promotes migration and invasion	Tumor differentiation, TNM stage, lymph node metastasis, inflammatory infiltration	(7, 8, 17, 48, 49)
Pancreatic cancer	IgG	87	HC48, SW1990, AsPC-1, BxPC-3, MIA PaCa-2, PANC-1, T3M4, CFPAC-1, HPAF	Inhibits the cytotoxic activity of NK cells, inhibits apoptosis, promotes growth, promotes migration and invasion, induces inflammation	Tumor differentiation, chemoresistance, metastasis, prognosis	(7, 23–25, 50)
Liver cancer	IgG	NA	BCL-7402, HepG2, Hep3B, Hep-2	Promotes growth, promotes migration, inhibits apoptosis	NA	(8, 18, 51, 52)
Gastric cancer	IgG	44	MGC-803, MKN28, AGS, BGC-823, SGC-7901	Promotes growth, promotes migration and invasion	Prognosis	(34, 45)
Esophageal cancer	IgG, Igκ, Igλ	77	Eca109, SHEEC	NA	Tumor differentiation, correlation with Ki67	(33)
Cervical cancer	IgG, IgA	NA	HeLa, C-33A, CA33, ME-180	Promotes growth, inhibits NK cell effector function, induces inflammation	NA	(8, 9, 18, 45, 52–54)
	Igκ	78	HeLa	Promotes malignant transformation	NA	(8, 9, 18)
Ovarian cancer	IgG	NA	CaOV3, SK-OV-3, OC-3-VGH	Promotes growth, promotes migration and invasion	NA	(8, 55)
Prostate cancer	IgG, Igκ	92	PC3, DU145, LNCaP	Promotes growth, promotes invasion and migration, inhibits apoptosis	Tumor differentiation	(18, 26–28, 56)
Bladder cancer	IgG	75-91	T24, BIU-87, 5637, EJ	Promotes growth, promotes migration and invasion, inhibits apoptosis	Tumor differentiation, recurrence	(36, 57)
Renal cancer	IgG	90	786-O, ACHN, Caki-1, 293	Promotes growth, promotes migration and invasion, inhibits apoptosis	Tumor differentiation, clinical stage, prognosis	(35, 58)
Nasopharyngeal cancer	IgA, Igκ	NA	CNE1, HNE2	Promotes growth, promotes malignant transformation	NA	(10, 22, 54)
Laryngeal cancer	IgM	63	HEp2	NA	Lymph node metastasis, clinical stage, prognosis	(7, 21)
Oral cancer	IgG, IgA	86	WSU-HN6, CAL27	Promotes growth, migration and invasion, inhibits apoptosis	NA	(29, 30)
Salivary gland cancer	IgG	52-60	SACC-83	Promotes growth, mediates motility, regulates EMT	Nerve invasion, metastasis, prognosis	(58)
Thyroid cancer	IgG	80	NA	NA	Differential diagnosis, lymph node metastasis	(31)
Parathyroid cancer	IgG	78	NA	NA	Differential diagnosis, recurrence, prognosis	(32)
Soft tissue tumor	IgG	97	A673, U-2OS, HT1080	NA	Tumor differentiation	(37, 38)
Acute myeloid leukemia	IgG	79	HEL, NB4, HL-60, OCI-AML3, THP-1	Promotes growth, inhibits apoptosis	Tumor differentiation, prognosis	(39, 42)
	IgM	50	THP-1, OCI-AML3, HL-60, U937, HEL, KG-1, NB4	Promotes growth	NA	(40)
	Igκ	94	HEL, HL-60, KG-1, NB4, OCI-AML3, THP-1	Promotes migration	NA	(41)

NA, not available. The presence of NA in the table is due to the absence of further studies.

### Characteristics of the Variable Region of Cancer-Derived Ig

V(D)J rearrangement is a process required for Ig expression and is initiated by recombination activating 1 (RAG1) and recombination activating 2 (RAG2) proteins. These two essential enzymes cooperate to generate double-strand breaks at recombination signal sites (RSSs), and the cleaved ends are joined through processes involving the end joining DNA repair system (59, 60). Although RAG1 and RAG2 were previously considered lymphoid-specific proteins, similar to cancer-derived Ig, these proteins have been detected in cancer cells (8, 18). Zheng et al. (61) sequenced 89 V_H_DJ_H_ transcripts from cancer cells purified by laser capture microdissection from eight different types of epithelial cancers and cells from two cancer cell lines (HT-29 and HeLa). The results suggested that the V_H_DJ_H_ transcripts of cancer-derived Ig share some features with Ig derived from B lymphocytes, such as V(D)J recombination, N-region insertion, and a high mutation rate. However, the V_H_DJ_H_ transcripts of cancer-derived Ig also show several characteristics distinct from those of B cell-derived Ig. First, in contrast to the highly variable sequences of classical Ig, the V_H_DJ_H_ transcripts of cancer-derived Ig present restricted V_H_DJ_H_ sequences. The V_H_DJ_H_ transcripts of cancer cells from one tumor sample or even different tumor samples possess identical V_H_DJ_H_ recombination patterns, junctions, and V region mutations. For example, V_H_5-51/D3-9/J_H_4 was repeatedly detected in half of the evaluated cases (19/38) of breast cancer, colon cancer, lung cancer, and oral cancer. In addition, cancer-derived IgG production in cancer cells might not follow CSR mechanisms. No identical patterns of V_Hγ_D_γ_J_Hγ_ and V_Hμ_D_μ_J_Hμ_ were detected in the study, indicating that IgM-producing cancer cells might not be the precursors of IgG producers. Subsequently, several unique V_H_DJ_H_ recombination patterns, such as V_H_3-33/D6-19/J_H_5 in oral cancer (29), V_H_3-48/D4-7/J_H_4 in acute myeloid leukemia (39), V_H_3-30/D6-6/J_H_4 in bladder cancer (57), V_H_3-7/D3-22/J_H_5 in colon cancer (48), and V_H_5-51/D3-16/J_H_4 in pancreatic cancer (25), have been identified.

### Cancer-Derived Ig Exhibits Aberrant Glycosylation

In carcinogenesis, glycans play important roles in cellular communication, cancer cell migration and invasion, cell-matrix interactions, metastasis formation and immune modulation (62). Several glycoconjugates, such as CA19-9, CEA, and CA125, have been applied clinically as tumor biomarkers (63). Glycosylation can profoundly regulate the physiochemical and biological properties of Ig molecules (64). For example, *N*-glycosylation at asparagine 297 (Asn297) is a consensus glycosylation event responsible for maintaining the effector functions of IgG (65). RP215 is a monoclonal antibody developed to recognize a glycan-associated epitope specifically expressed in cancer cells (66). Studies have shown that the antigen recognized by RP215 is an IgG molecule with aberrant glycosylation expressed by cancer cells (67). The RP215-recognized glycosylation of cancer-derived IgG was further revealed to be N-glycosylation at asparagine 162 (Asn162), located in the CH1 domain of the IgG heavy chain and carrying a sialic acid modification (44, 68). In subsequent studies, RP215 showed more specificity than commercial anti-IgG antibodies in discriminating cancer-derived IgG from B cell-derived IgG due to its capacity to recognize the specific sialylation site of cancer-derived Ig (25, 69).

## Regulatory Mechanisms of Ig Expression in Cancer Cells

The transcription of the Ig gene depends on the activation of Ig promoters and enhancers, which requires the participation of multiple transcription factors such as E2A, EBF, Pax5 and Oct-2 (70–72). Since Ig was previously thought to be selectively expressed in B lymphocytes, how Ig transcription is regulated in cancer cells is unknown. The Ig transcription factors E2A and Oct-1 were first found to be widely expressed in cancer cell lines (73). Using a dual-luciferase reporter assay, the 5’-flanking sequence of V_H_4-59 was revealed to present promoter activity in cancer cell lines. An enhancer-like element and a copromoter-like element were identified, and the octamer element (ATGCAAAT) was shown to be crucial for Ig gene transcription in cancer cells. In contrast to B cells, which utilize the Ig transcription factor Oct-2, cancer cells tend to utilize Oct-1. Similarly, the 1,200-bp fragment upstream of V_H_6-1 was also identified to exhibit promoter activity in cancer cells (74). Additionally, the IgIα1 promoter was found to be activated by the transcription factor Ets-1 in cancer cells (75). Other Ig transcription factors, including EBF and Pax5, have also been detected in cancer cells (76). The above studies initially describe how cancer-derived Ig is regulated at the transcriptional level.

In addition to internal Ig transcription factors, several external factors, such as pathogen infection or cytokine stimulation, can regulate Ig expression in cancer cells. Epstein-Barr virus (EBV)-encoded latent membrane protein 1 (LMP1) is an EBV latent gene that plays important roles in carcinogenesis. Previous researchers found that the expression of Igκ in LMP1-positive nasopharyngeal cancer cells was significantly higher than that in LMP1-negative cells (77). Further investigations revealed that the Igκ transcription factors NF-κB (p52/p65) and AP-1 (c-Jun/c-Fos) could be stimulated by LMP1 and then bind the Igκ gene enhancer iEκ, resulting in the upregulation of Igκ expression (78). Merkel cell carcinoma (MCC) is a type of skin cancer that can be divided into the following two types: Merkel cell polyomavirus (MCPyV)-positive MCC and MCPyV-negative MCC. Ig is expressed in 70% of MCPyV-positive MCCs, while no MCPyV-negative MCCs have been shown to express Ig, suggesting that Ig expression in MCC may be induced by MCPyV infection (79). Furthermore, Toll-like receptor 9 (TLR9) agonist stimulation, which mimics bacterial infection, can increase the secretion of IgM *via* the TLR9-MyD88 pathway (16). The above phenomena imply that pathogen infection may participate in regulating the expression of cancer-derived Ig. In addition, several crucial cytokines, including TGF-β and TNF-α, have been proven to regulate Ig expression in cancer cells in a dose-dependent manner (75, 80). A comparison of the characteristics of cancer-derived Ig and B cell-derived Ig is provided in **Table 2**.

**Table 2:** Comparison between the characteristics of cancer-derived Ig and B cell-derived Ig.

	Cancer-derived Ig	B cell-derived Ig
Expression profile	Cancer cell	B cell
Function	Promotes tumorigenesis Promotes tumor immune escape Induces inflammation Activates platelet aggregation	Antibody activity B cell development
Recombination patterns of variable region	Restricted	Highly variable
N-Glycosylation site	Asn162	Asn297
Transcription factor	Oct-1	Oct-2

## Cancer-Derived Ig Performs Critical Roles in Cancer Progression

The functions of cancer-derived Ig are distinct from those of B cell-derived Ig, which exerts antibody functions in humoral immunity. Studies have shown that cancer-derived Ig acts as a crucial protumorigenic molecule that promotes carcinogenesis *via* several different mechanisms.

### Cancer-Derived Ig Promotes Malignant Behaviors in Cancer Cells

Cancer-derived Ig acts as a growth factor-like molecule that directly promotes the malignant behaviors of cancer cells including proliferation, migration, invasion, and apoptosis resistance. The protumorigenic effect of cancer-derived Ig on cancer cells has been confirmed in multiple different types of cancers (20, 25, 39, 54). The blockade of cancer-derived IgG by antisense DNA or a specific antibody profoundly suppresses the growth of cancer cells (8, 68). Furthermore, cancer-derived IgG is highly expressed in basal-like cancer cells and cancer cells at the tumor boundary. After sorting different populations of cancer cells based on the IgG expression level, cancer cells with high IgG expression display cancer stem cell-like properties, such as the coexpression of CD44v6, a high sphere-forming capability and resistance to chemotherapy (69). To elucidate the molecular mechanisms of action involving cancer-derived Ig, the major downstream pathways that cancer-derived Ig may regulate have been further studied. Twenty-seven potential IgG-interacting proteins were previously identified by a coimmunoprecipitation assay in cancer cells (81). Among the identified proteins, RACK1, RAN, and PRDX1, which are closely associated with cell growth and oxidative stress, were confirmed to interact with IgG. The induction of intracellular reactive oxygen species (ROS) was further revealed to be an important pathway regulated by cancer-derived IgG. Cancer-derived IgG also interacts with many membrane proteins involved in cell-cell adhesion junctions, focal adhesion and hemidesmosomes (69). For example, cancer-derived IgG can be secreted in autocrine manners by cancer cells, specifically interact with the integrin α6β4 complex and subsequently activate the FAK-Src pathway, which is a critical downstream pathway of integrins (68, 82). The protumorigenic roles of secreted cancer-derived IgG can be blocked by its specific monoclonal antibody RP215. In another study, the MEK/ERK/c-Myc pathway was demonstrated to be another downstream pathway of cancer-derived IgG regulating cell growth and the cell cycle (83). Moreover, Igκ and Igλ have been shown to be responsible for maintaining high expression of the antiapoptotic molecule Bcl-xL and thus perform roles in resisting apoptosis (84).

### Cancer-Derived Ig Promotes Tumor Immune Escape

Ig is well known for mediating a wide range of effector functions that modulate several aspects of innate and adaptive immunity (85). Modified by given glycosylation, the functions of Ig can dramatically switch from immunoreactive roles to immunosuppressive roles (86). For example, it has been reported that the anti-inflammatory effect of intravenous immunoglobulin (IVIG) depends on a small fraction of sialylated IgG (87). These sialylated IgG molecules can inhibit the functions of dendritic cells (DCs) and CD4^+^ T cells by binding the sialic acid receptor DC-SIGN on DCs. Considering that cancer-derived Ig is not only distributed in the cytoplasm but is also located on the cell membrane and secreted, it could be significant to explore whether cancer-derived Ig molecules can modulate other cell types, such as immune cells. Wang et al. (44) purified cancer-derived IgG from the tumor microenvironment and identified a large fraction of sialylated cancer-derived IgG (SIA-CIgG). Using *in vitro* and *in vivo* models, these authors demonstrated that SIA-CIgG could significantly inhibit T cell proliferation and reduce effector T cell frequencies in a dose-dependent manner. As SIA-CIgG was purified from the general tumor microenvironment, the immunosuppressive effect of SIA-CIgG may attribute to both membrane-bound and secreted cancer-derived Ig. Further experiments are needed to elucidate whether membrane-bound and secreted cancer-derived Ig share the same function. Furthermore, the inhibitory effect of SIA-CIgG on effector T cells was revealed to rely on its binding with sialic acid-binding immunoglobulin-type lectins (Siglecs). The immunosuppressive function of SIA-CIgG significantly depends on its sialyation modification at the Ans162 site. Siglecs are found on most immune cells and have a common N-terminal domain that recognizes sialic acid-containing glycans (88). Recent studies have shown that Siglecs are highly expressed in tumor infiltrating immune cells and serve as immune checkpoints regulating anti-cancer immunity (89, 90). However, the ligands of Siglecs in tumor immunity are largely unknown. Thus, SIA-CIgG is considered a ligand of Siglec, and combined, they may serve as a pair of potential immune checkpoint molecules mediating tumor immune escape. Targeting SIA-CIgG/Siglecs with specific antibodies could be a promising approach to reversing immune suppression in cancer. In addition, cancer-derived Ig can downregulate the cytotoxic activity of natural killer (NK) cells *via* the inhibition of ADCC function (23, 45). The presence of cancer-derived Ig specifically reduces the ADCC effect induced by anti-human epithelial growth factor receptor (EGFR) antibodies in a dose-dependent manner.

### Other Mechanisms of Cancer-Derived Ig Involved in Promoting Carcinogenesis

**Figure 1:**
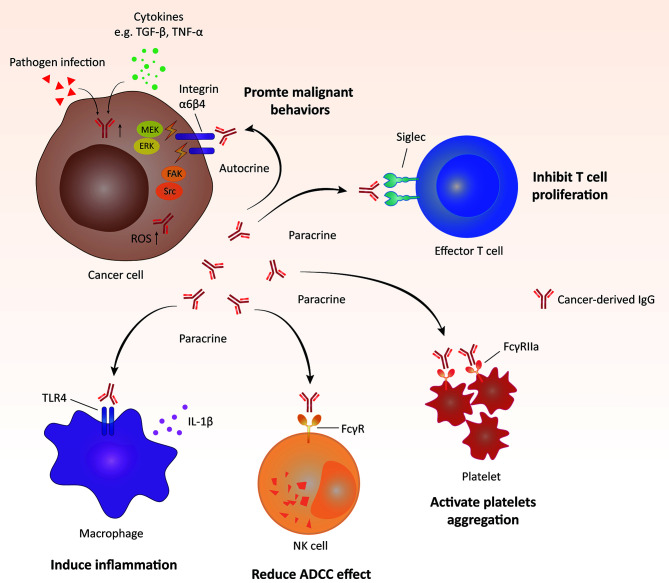
Molecular mechanisms of action of cancer-derived IgG. Cancer-derived IgG promotes tumorigenesis in autocrine and paracrine manners.(i) Cancer-derived IgG interacts with integrins located on the tumor cell membrane and then activates downstream pathways, including FAK/Src, MEK/ERK/c-Myc and reactive oxygen species (ROS) pathways. (ii) Cancer-derived IgG interacts with different membrane receptors of other types of cells, including Siglec of T cells, Toll-like receptor (TLR) of macrophages and FcγR of natural killer (NK) cells and platelets, and further regulates the functions of these cell types. ADCC, antibody-dependent cellular cytotoxicity.

Cancer-derived Ig also plays important roles in the regulation of protumorigenic inflammation. After stimulation with lipopolysaccharide (LPS), cancer-derived IgG expression is significantly upregulated. A reduction in the IgG level can directly downregulate Toll-like receptor 4 (TLR4) expression, resulting in the suppressed phosphorylation of NF-κB and MAPK and attenuated production of LPS-induced proinflammatory cytokines (53). In addition, cancer-derived IgG is identified as a functional component of pancreatic cancer cell debris that can induce inflammation by stimulating IL-1β release from tumor-associated macrophages *via* the TLR4/TRIF/NF-κB pathway (50). Furthermore, free Ig light chain (FLC) exerts an essential promotive effect on colitis-associated colon carcinogenesis by activating the inflammasome (91). The FLC blocker F991 can significantly impede the process of colon carcinogenesis by inhibiting the activation of the inflammasome and reducing the levels of cleaved caspase-1, IL-1β and IL-18. Additionally, cancer-derived IgG has the ability to activate platelets and, thus, participates in tumor-associated thrombosis (92). This effect is mediated by the interaction between cancer-derived IgG and platelet FcγRIIa, which upregulates the expression of CD62P and PAC-1, platelet aggregation, and ATP release. The above molecular mechanisms of action of cancer-derived IgG are summarized in **Figure 1**.

## Clinical Significance of Cancer-Derived Ig in Cancer

With the identification of the critical roles of cancer-derived Ig in tumorigenesis, studies have begun to explore whether cancer-derived Ig can serve as a promising target for clinical applications. The expression of cancer-derived IgG is found to be closely associated with tumor differentiation in colon cancer (17), lung cancer (20), renal cancer (35), and pancreatic cancer (25, 93). The expression of cancer-derived Ig is also related to cancer metastasis. The potential of a high expression of cancer-derived IgG to act as a predictor of lymph node metastasis has been indicated in breast cancer (15), lung cancer (46), colon cancer (17), and thyroid cancer (31). Furthermore, cancer-derived Ig can serve as a promising prognostic factor predicting worse outcomes in patients with lung cancer (20), pancreatic cancer (25), gastric cancer (34), renal cancer (34), salivary gland cancer (94), and parathyroid cancer (32). The above studies indicate that cancer-derived Ig could be a diagnostic and prognostic marker in clinical applications in cancer patients. More importantly, cancer-derived Ig is specifically expressed in cancer cells, rendering it a potentially anti-cancer therapeutic target. Specific antibodies targeting cancer-derived IgG have shown promising anti-cancer therapeutic effects in preclinical models (44, 68). Considering its localization on cancer cell membranes, a new generation of chimeric antigen (CAR)-T cell targeting cancer-derived IgG is also worth exploring. Future translational studies and clinical trials are needed to evaluate whether cancer-derived Ig could serve as a novel target or joint target for cancer therapy. Moreover, cancer-derived Ig can be detected by the RP215 antibody in the serum of cancer patients and shows considerable value in monitoring cancer progression (95). Recently, serum IgG glycosylation has been identified as having great significance in differential diagnosis, disease progression monitoring, and therapeutic efficacy prediction in cancer patients (96–100). Similarly, autoantibodies have also been considered next generation biomarkers of cancers (101–104). It will be interesting to perform more studies to elucidate whether these serum aberrant glycosylated Ig or autoantibodies are secreted by cancer cells and evaluate whether secreted cancer-derived Ig can serve as a valuable circulating biomarker in cancer patients.

## Conclusions and Perspectives

Ig is a well-known molecule essential for the humoral immune response, but its roles in tumorigenesis are far from clear. Some studies have elucidated the effects of Ig on B cell infiltration in the tumor microenvironment, but many phenomena are still difficult to understand from the perspective of B cell-derived Ig (105–107).

The identification of cancer-derived Ig is undoubtedly a significant finding in the current research area of Ig and largely improves the understanding of Ig in tumorigenesis. Ig is highly expressed in various types of malignancies and is produced by cancer cells. Cancer-derived Ig is located in the cytoplasm and on the cell membrane of cancer cells and can be secreted extracellularly. Compared to B cell-derived Ig, cancer-derived Ig presents distinct characteristics, such as restricted variable region sequences and aberrant glycosylation. Functionally, cancer-derived Ig performs protumorigenic roles *via* several different mechanisms. Moreover, preliminary studies have shown that cancer-derived Ig has the potential to serve as a novel diagnostic and therapeutic target in clinical applications.

Although the current results are promising, more work is needed to understand the roles of cancer-derived Ig in tumorigenesis. First, the variable region of Ig is essential for recognizing diverse antigens based on its specific sequence. Several molecules have been identified to interact with cancer-derived Ig (24, 89). Considering the restricted patterns of the variable region of cancer-derived Ig, it could be critical to identify the types of molecules that interact with cancer-derived Ig and the specific functions of the variable regions of cancer-derived Ig. Second, cancer-derived Ig has shown pivotal modulatory effects on immune cells, including NK cells and T cells, supporting its function as an immune checkpoint molecule. More studies are needed to elucidate the roles of cancer-derived Ig in antitumor immunity. Finally, it could be important to explore the significance of cancer-derived Ig in clinical applications. Previous studies have demonstrated that cancer-derived Ig can serve as a promising diagnostic and prognostic biomarker in cancer patients. Therapies targeting cancer-derived Ig have shown promising effects in preclinical studies. Future studies are expected to evaluate whether it is feasible to apply cancer-derived Ig as a therapeutic target in cancer patients.

## Author Contributions

MC and JH drafted the manuscript. SZ and QFL collected related literature. XQ and QL directed the work and revised the manuscript. All authors contributed to the article and approved the submitted version.

## Funding

This work was supported by grants from the National Natural Science Foundation of China (81872501 and 81673023), the Key Project of the National Natural Science Foundation of China (82030044), the Key Support Projects of the National Natural Science Foundation’s Major Research Program (91642206), and the Non-profit Central Research Institute Fund of the Chinese Academy of Medical Sciences (2018PT32014).

## Conflict of Interest

The authors declare that the research was conducted in the absence of any commercial or financial relationships that could be construed as a potential conflict of interest.
