# Supplementary material for: Immunoglobulin Expression in Cancer Cells and Its Critical Roles in Tumorigenesis
Source: Front Immunol. 2021 Mar 24;12:613530. doi: 10.3389/fimmu.2021.613530 (PMC8024581; doi:10.3389/fimmu.2021.613530)
Supplement: Supplementary file 1 [file DataSheet_1.docx]

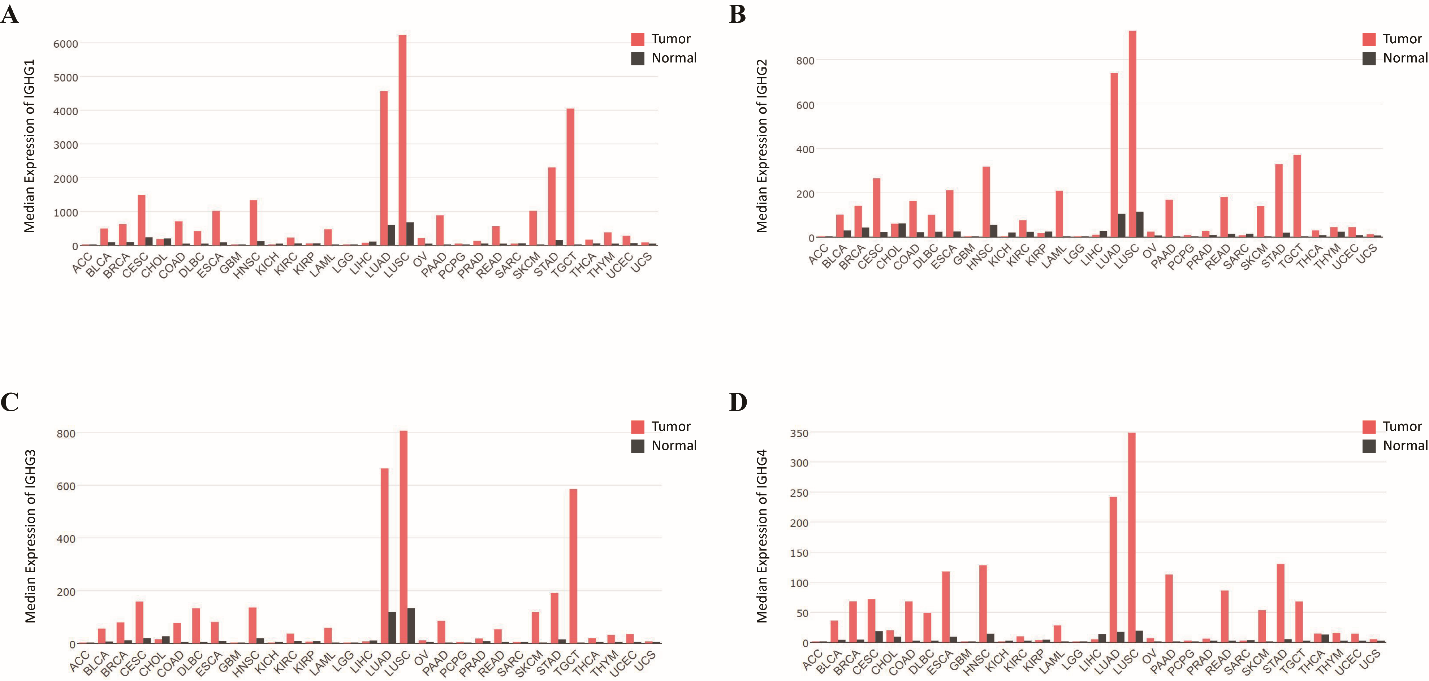


**Supplementary Figure 1.** The expression levels of IgG are significantly elevated in malignancies. The expression levels of IGHG1 (**A**), IGHG2 (**B**), IGHG3 (**C**) and IGHG4 (**D**) are generally overexpressed in various types of tumor tissues compared with the corresponding peritumoral tissues in the GEPIA database.
